# Evaluating the compressive strength of glass powder-based cement mortar subjected to the acidic environment using testing and modeling approaches

**DOI:** 10.1371/journal.pone.0284761

**Published:** 2023-04-24

**Authors:** Majdi Ameen Alfaiad, Kaffayatullah Khan, Waqas Ahmad, Muhammad Nasir Amin, Ahmed Farouk Deifalla, Nivin A. Ghamry

**Affiliations:** 1 Department of Chemical Engineering, College of Engineering, King Faisal University, Al-Ahsa, Saudi Arabia; 2 Department of Civil and Environmental Engineering, College of Engineering, King Faisal University, Al-Ahsa, Saudi Arabia; 3 Department of Civil Engineering, COMSATS University Islamabad, Abbottabad, Pakistan; 4 Department of Structural Engineering and Construction Management, Future University in Egypt, New Cairo City, Egypt; 5 Faculty of Computers and Artificial Intelligence, Cairo University, Giza, Egypt; Mirpur University of Science and Technology, PAKISTAN

## Abstract

This study conducted experimental and machine learning (ML) modeling approaches to investigate the impact of using recycled glass powder in cement mortar in an acidic environment. Mortar samples were prepared by partially replacing cement and sand with glass powder at various percentages (from 0% to 15%, in 2.5% increments), which were immersed in a 5% sulphuric acid solution. Compressive strength (CS) tests were conducted before and after the acid attack for each mix. To create ML-based prediction models, such as bagging regressor and random forest, for the CS prediction following the acid attack, the dataset produced through testing methods was utilized. The test results indicated that the CS loss of the cement mortar might be reduced by utilizing glass powder. For maximum resistance to acidic conditions, the optimum proportion of glass powder was noted to be 10% as cement, which restricted the CS loss to 5.54%, and 15% as a sand replacement, which restricted the CS loss to 4.48%, compared to the same mix poured in plain water. The built ML models also agreed well with the test findings and could be utilized to calculate the CS of cementitious composites incorporating glass powder after the acid attack. On the basis of the R^2^ value (random forest: 0.97 and bagging regressor: 0.96), the variance between tests and forecasted results, and errors assessment, it was found that the performance of both the bagging regressor and random forest models was similarly accurate.

## 1. Introduction

When measured against annual use, cement-based materials are second only to water in terms of worldwide consumption [1–3]. Cement-based materials derivatives are used extensively in the building because of their affordability and durability [4–6]. Retaining their quality, mechanical performance, and functionality after being exposed to external conditions is a hallmark of durable cement-based materials [7–9]. Cement-based material’s durability is measured by how long it lasts while being subjected to chemical attacks, abrasion, weathering, and other forms of deterioration [10–12]. When exposed to certain corrosive elements, cement-based materials deteriorate. The attacking mechanism might be either internally or externally sourced and could be physical, mechanical, or chemical in nature. The aggregate and paste of composites can be damaged by a variety of physical and chemical attacks [13]. Cement-based material’s strength and durability performance diminish when subjected to several types of harmful agents [14], making their performance in a hostile environment the primary issue. Acid, sulfate, salts, and other hazardous compounds, as a result of the fast growth of the industrial sector, regularly attack cement-based materials. The fundamental goal of the construction sector right now is to create a building material that can survive harsh weather conditions and satisfy durability standards [15].

One of the most crucial aspects influencing the durability of cement-based materials is their resistance to the invasion of hostile ions. Indirectly, the absorbency of cement-based materials, the permeable pore volume inside the composites, and the connectivity between pores all reflect their porosity [16]. One of the most destructive acids that deteriorate cement-based materials is sulfuric acid (H_2_SO_4_) [17]. A cement-based concrete structure under attack by H_2_SO_4_ may suffer extensive damage and deterioration, leading to premature collapse. Alkaline pore water found in cement-based materials combines with the calcium-silicate-hydrate (CSH) gel and Ca(OH)_2_ in cement paste to produce dissolved ions, which then dissolve the material’s constituents when exposed to moderate or strong acids [14]. Since the attack of H_2_SO_4_ is coupled with sulfate, it is one of the most harmful acids for cement-based materials [17]. Sulfate attack is a prominent component in the deterioration of cement-based materials and affects their durability because of the chemical assault by sulfates and the physical attack owing to salt crystallization [18, 19]. The breakdown of CSH leads to the formation of additional compounds, which contribute to the durability issues of cement-based materials [18]. Hence, it is important to study how acid attacks could shorten the lifespan of cement-based materials.

Cement manufacturing requires considerable energy and produces significant carbon dioxide emissions, which contribute to global warming [20]. Cement manufacturers may cut costs and carbon dioxide emissions by recycling and reusing waste [21–24]. Therefore, there is a critical demand for eco-friendly cement-based materials in the construction industry [25–28]. Moreover, the extraction of natural aggregates releases a considerable proportion of carbon dioxide and causes the depletion of natural resources [29]. In the construction industry, recycled glass powder (RGP) modified cement-based materials are rising in popularity due to their low cost and widespread availability [30]. Mechanical properties, such as compressive and flexural strength, are improved by replacing 10–20% cement or sand with RGP [31, 32]. As a result, replacing sand with RGP will help save natural resources and make waste management easier, and its usage as a cement substitute will aid in lowering cement demand and carbon dioxide emissions [33, 34].

Experts are developing prediction models for the performance of materials and buildings in an effort to cut down on repetitive lab tests [35–39]. Prediction models, such as regression-based techniques, are used to estimate the material’s attributes [40–42]. In this context, artificial intelligence (AI) approaches like machine learning (ML) represent the cutting edge of model development [43–45]. There has been a rise in the use of ML methods for forecasting building materials performance [46–48]. Only a few articles have dealt with predicting the features of cement-based materials modified with RGP [32, 49, 50], while most earlier ML research has focused on the strength of traditional cement-based materials [51–53]. However, no study was found in the literature for the prediction of CS of cement-based materials containing RGP after the acid attack using ML methods.

In this study, experimental and ML techniques were used to assess CS variation in RGP-modified cement mortar after the acid attack. Various amounts of RGP were employed in place of the traditional sand and cement to make the mortar samples. The data sample collected from the experimental strategy was utilized to develop ML estimation models. The objectives of the study were satisfied by employing ensemble ML techniques, including bagging regressor and random forest. Due to the superior performance of ensemble ML approaches compared to those of individual ML algorithms, only ensemble methods were employed in this research. Multiple measures, including the coefficient of determination (R^2^), statistical tests, the k-fold approach, and divergence of predicted results, were used to evaluate the efficacy of ML algorithms. This investigation is intriguing since it employs both experimental and ensemble ML techniques to assess the CS variation subsequent to an acid attack of cement mortar containing RGP. The goal of this study was to learn more about ML techniques for making accurate predictions of material characteristics. A dataset is necessary for applying ML methods, which can be compiled by experimentation or mining existing data sources. To approximate material properties, the acquired data set might be fed into ML models. With seven input variables and experimental data, ML techniques were evaluated to see how well they could predict the variation in CS after the acid attack on cement mortar containing RGP. The significance of raw components was further explored using SHapley Additive exPlanations (SHAP) analysis. SHAP employs intricate non-linear actions, a broad spectrum of input component effects, and a weighting characteristic for each input in an effort to provide a nuanced comprehension of RGP-containing cement mortar construction.

## 2. Methods

### 2.1 Materials and testing

Sand and Portland cement were acquired from a regional supplier, and silica fume and superplasticizer were procured from PAGEL Chemicals Pakistan. The glass waste was collected from local construction debris, brought to the lab, cleaned, mechanically crushed into powder, and sieved through a #200 sieve. Three mix proportions were chosen for preparing cement mortar samples. The cement-to-sand ratio was maintained at 1:1, the water-to-cement ratio was maintained at 0.25, and the superplasticizer dose was maintained at 4% by mass of cement in all the mixes. The quantity of silica fume (SF) was the sole distinguishing factor amongst the mixtures. The percentage of SF in the cement in Mixes SF-15, SF-20, and SF-25 was 15%, 20%, and 25% by cement mass, respectively. In addition, cement and fine aggregate were replaced with RGP in all mixtures, with the percentages ranging from 0% to 15% in 2.5% increments. The components of the cement mortar were mixed together using a motorized mixer. The sand, cement, RGP, and silica fume were mixed for 2 minutes with half of the water containing the superplasticizer. The remaining water was added to the mixer in two parts, and the total time given for mixing the ingredients was four minutes.

**Fig 1:**
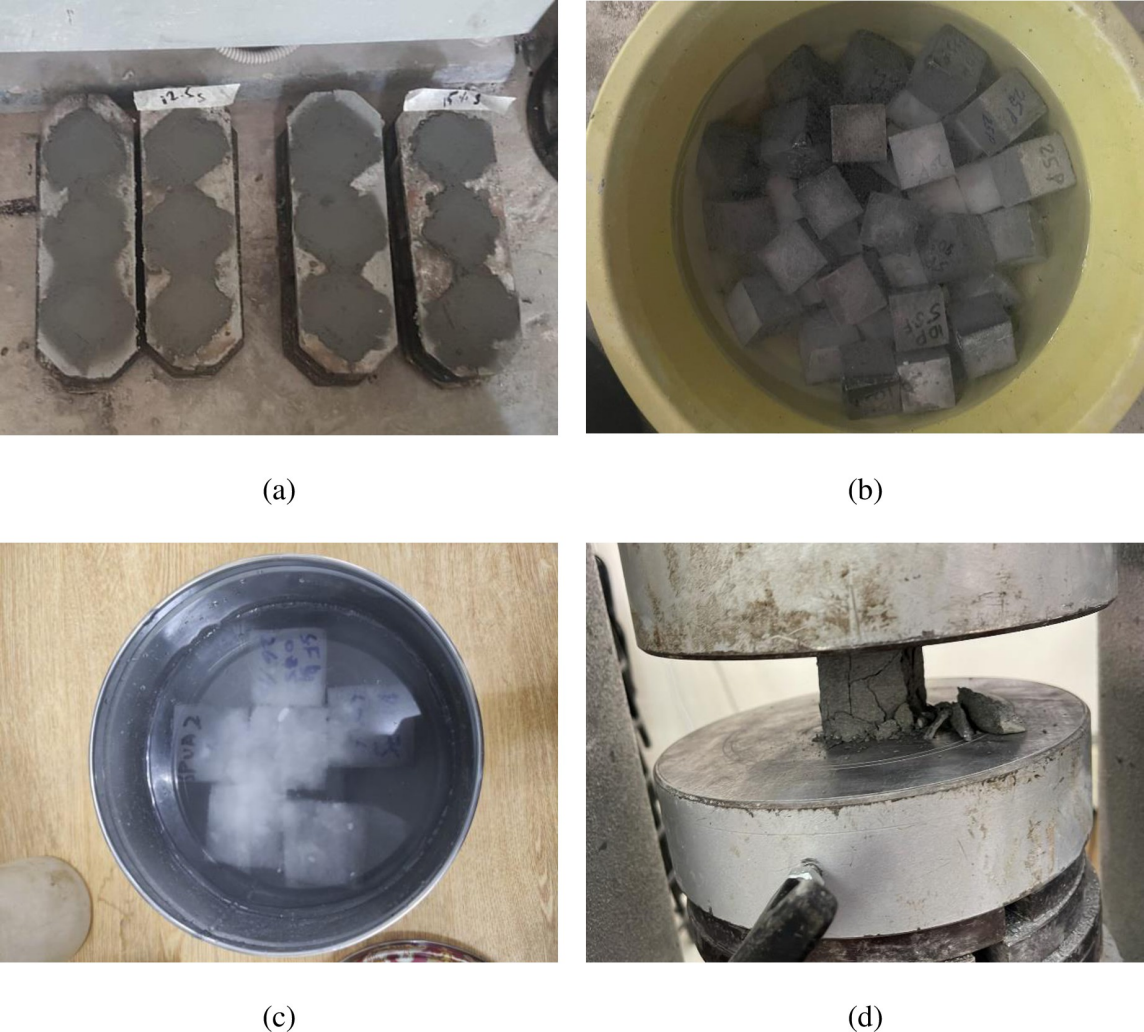
Experimental setup’s images: (a) Samples in molds; (b) Samples in plain water; (c) Samples in 5% H_2_SO_4_ solution; (d) Testing sample under uniaxial compression.

For the purpose of evaluating the CS, 50 mm cube samples were cast. For each mix design, six samples were cast, for a total of 234 samples. After being cast, samples were kept in molds for 24 hours before being demolded and cured for 28 days. To compare the effect of curing age on the strength of 90-day-old samples cured in an acidic environment to those aged in plain water, the samples were also immersed in plain water under normal conditions. The samples were immersed in a 5% H_2_SO_4_ solution, and the acidic solution’s pH was kept below 3. To maintain consistency, the acidic solution’s pH was periodically tested. All specimens were subjected to a uniaxial compression test in accordance with ASTM C109/C109M-20 [54] after 90 days. The percent reduction in CS of acidic samples relative to water-cured samples was measured. The final result for each proportion of the mixture in each group was derived by averaging the results of three readings. Fig 1 illustrates the experimental setup at different stages.

### 2.2 Machine learning-based modeling

**Fig 2:**
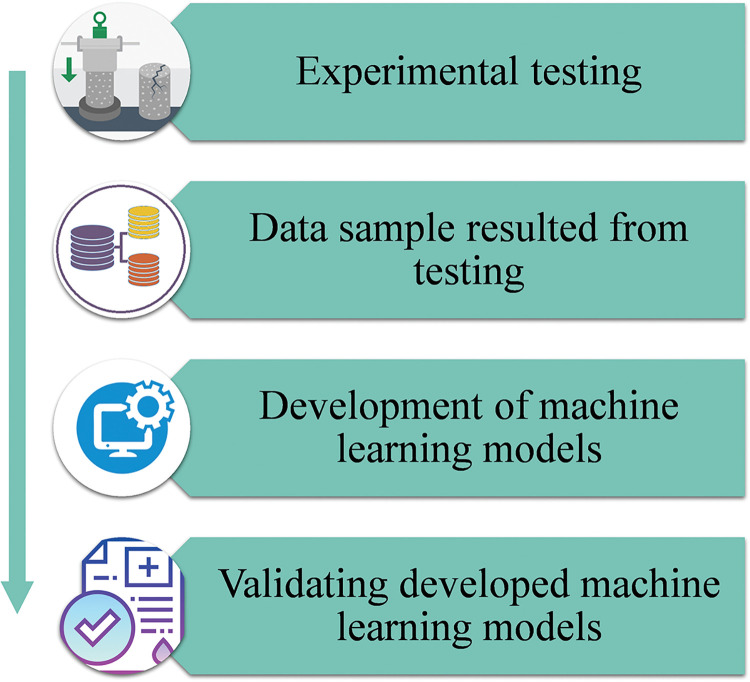
Flow diagram of research strategy.

A high number of input parameters is required for ML approaches to provide the desired result [55]. For the desired outcomes from an ML approach, the data sample’s parameters need to be continually changing; utilizing a constant value or one with limited variation may result in subpar outcomes [56, 57]. Using an experimental dataset, the CS of cement mortar modified with RGP following an acid assault was predicted. The ML techniques utilized cement, water, sand, 90-day CS, superplasticizer, RGP, and SF as inputs for predicting the CS after the acid attack. To do this, Python code in Spyder (5.2.2) from the Anaconda Navigator software was used to build ensemble ML models such as bagging regressor and random forest. The material properties may be correctly anticipated using ML techniques [58, 59]. For running the algorithms, 70% of the data was utilized for training and 30% for testing. The R^2^ value measures the degree of agreement between actual and expected results. The R^2^ number indicates the discordance between the predicted and real data [20]. Closer to 1 indicates less divergence, whereas closer to 0 indicates greater divergence. The model’s efficacy was evaluated using k-fold and statistical approaches. The statistical errors of the models were quantified and compared using a variety of measures, including mean absolute error (MAE), root mean square error (RMSE), and mean absolute percentage error (MAPE). Additionally, SHAP analysis was used to explore the relevance of raw components even deeper. Fig 2 is a flowchart illustrating the sequence of ML-based modeling. The subsequent segments detail the ML algorithms and validation methods applied in this research.

#### 2.2.1 Bagging regressor

**Fig 3:**
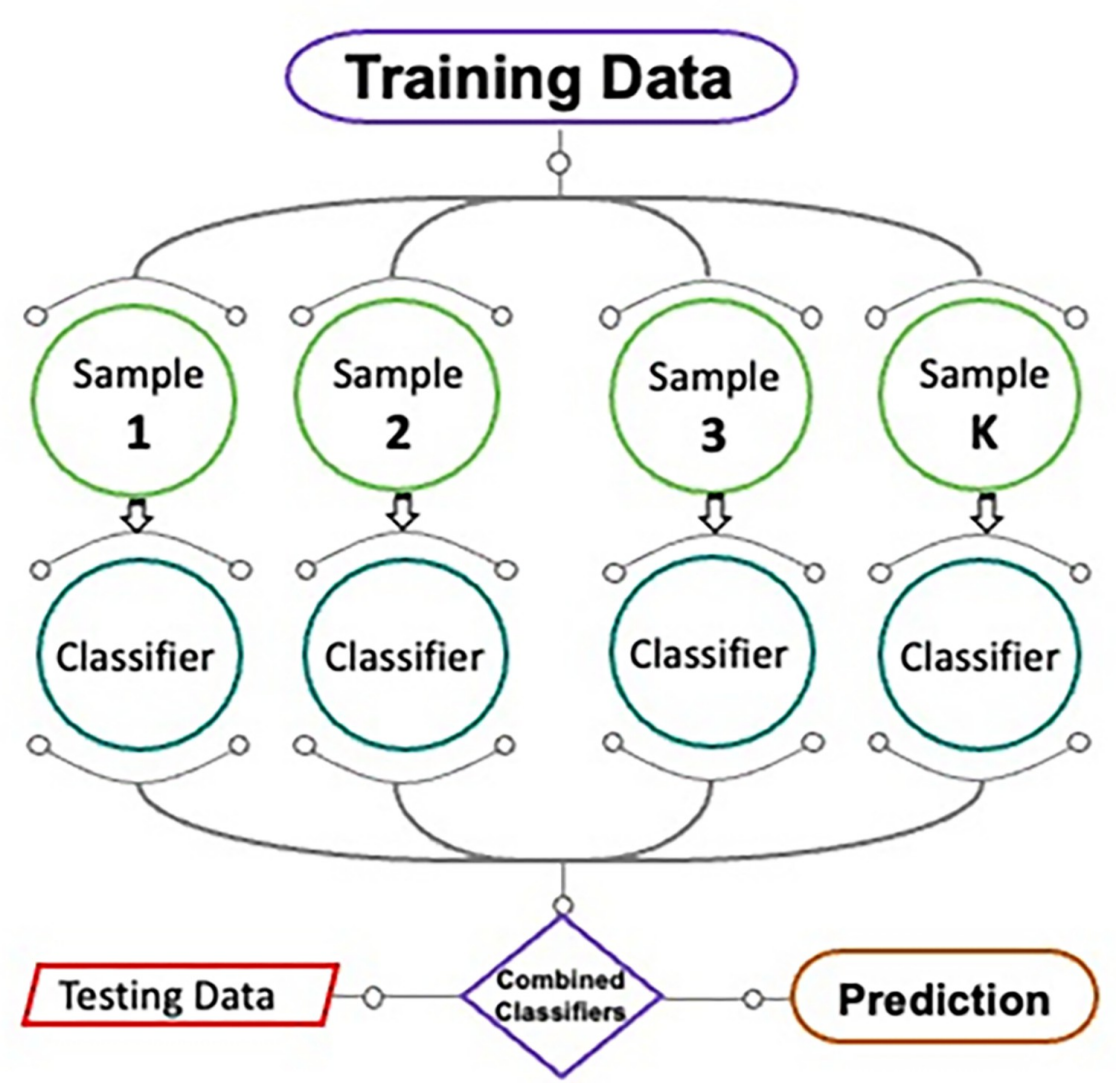
Process of bagging regressor training method [61].

In Fig 3, a simplified representation of the bagging regressor procedure is shown. The modification to the prediction model due to new training data also relies on an equivalent ensemble method. Information from the main data set is used as a stand-in in the random sampling method. In replacement sampling, it is possible for two or more observations to appear in separate samples of training data. Each component in the new data sample has the same likelihood of occurrence after bagging. It does not matter how much data is projected because that would not change the size of the training set. In addition, the divergence may be greatly minimized by training more models with these data samples and enhancing the estimation of the needed result. This ensemble uses the average of all model projections. In regression, a good estimate can be obtained by taking the mean of several different models’ predictions [60]. A decision tree is utilized to fine-tune the bagging technique, and a total of 20 submodels are employed to determine the most productive value in terms of output.

#### 2.2.2 Random forest

**Fig 4:**
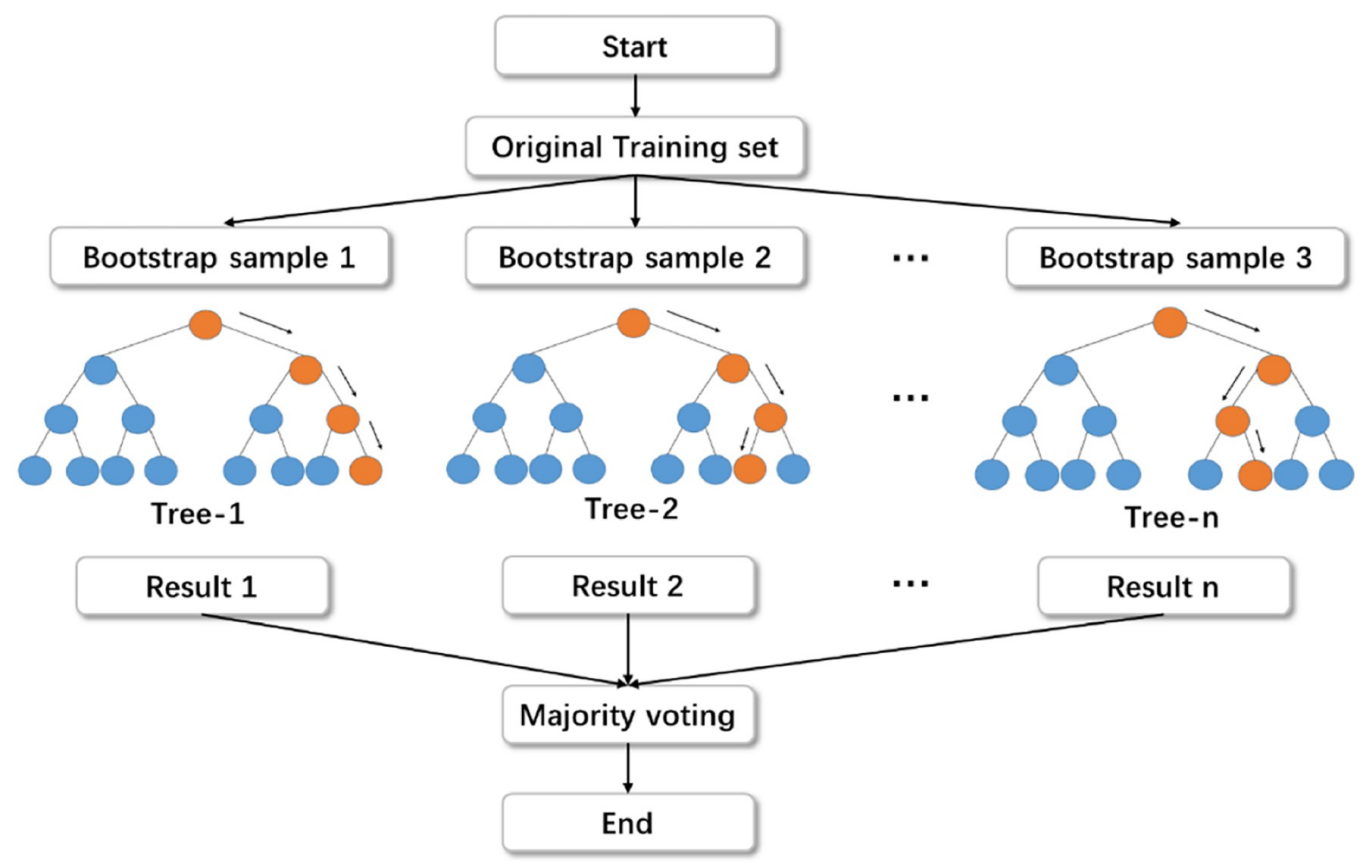
Process of random forest training method [63].

A random forest is a method for producing a group of regression trees with reduced variance. The decision trees group together to form a forest using the concept of “bootstrap aggregation” (bagging) to produce many similar data samples taken from the same source dataset. Bagging refers to the process of combining a basic model that has been trained with data used for training. Due to its small bias and considerable variance, the tree is prone to overfitting. One major advantage of the random forest method is how much instability it may reduce. Although the decision tree is capable of ML on its own, this independence comes at the cost of a tendency to overfit training data. Many decision trees and rules are only a few examples of the many methods available for generating random forest models without resorting to overfitting [62]. Since this was a controlled experiment, the model was managed. The random forest process’s flowchart is shown in Fig 4.

#### 2.2.3 Validation strategies

**Fig 5:**
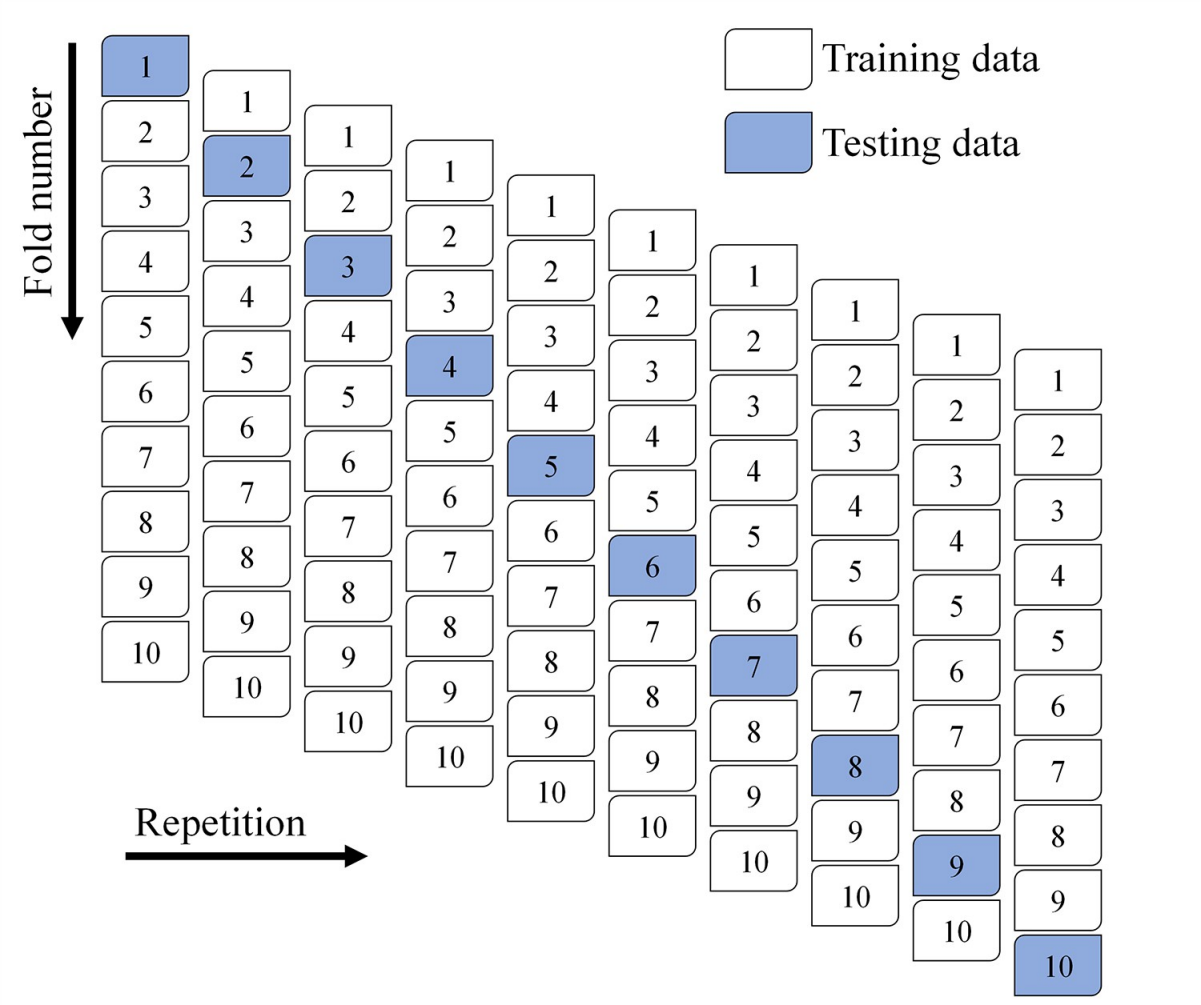
Schematic illustration of k-fold approach.

K-fold and statistical testing on the ML algorithms were used for validation. The k-fold technique, which randomly divides the dataset into 10 classes, is often used to assess a method’s performance [64]. The k-fold procedure uses nine-class training data and single-class testing data [32], as depicted in Fig 5. An accurate ML model will have lower error rates and a higher R^2^ value. To get the most out of this strategy, the process was repeated ten times. The approach is largely responsible for the model’s high degree of accuracy. Statistical error analysis (MAE, RMSE, and MAPE) was also used to compare the efficacy of different ML methods. The statistical performance of the ML models’ forecasts was analyzed using Eq (1)–(3), which were acquired from previous work [65, 66].

$$MAE=\frac{1}{n}\sum_{i=1}^{n}\left|P_{i}-Ei\right|,$$ (1)

$$RMSE=\sqrt{\sum \frac{{\left(P_{i}-E_{i}\right)}^{2}}{n}},$$ (2)

$$MAPE=\frac{100\%}{n}\sum_{i=1}^{n}\frac{\left|P_{i}-Ei\right|}{E_{i}},$$ (3)

where *n* = number of data points, *P_i_* = estimated results, and *E_i_* = actual results.

## 3. Results and analysis

### 3.1 Influence of RGP on 90-day CS in plain water

**Fig 6:**
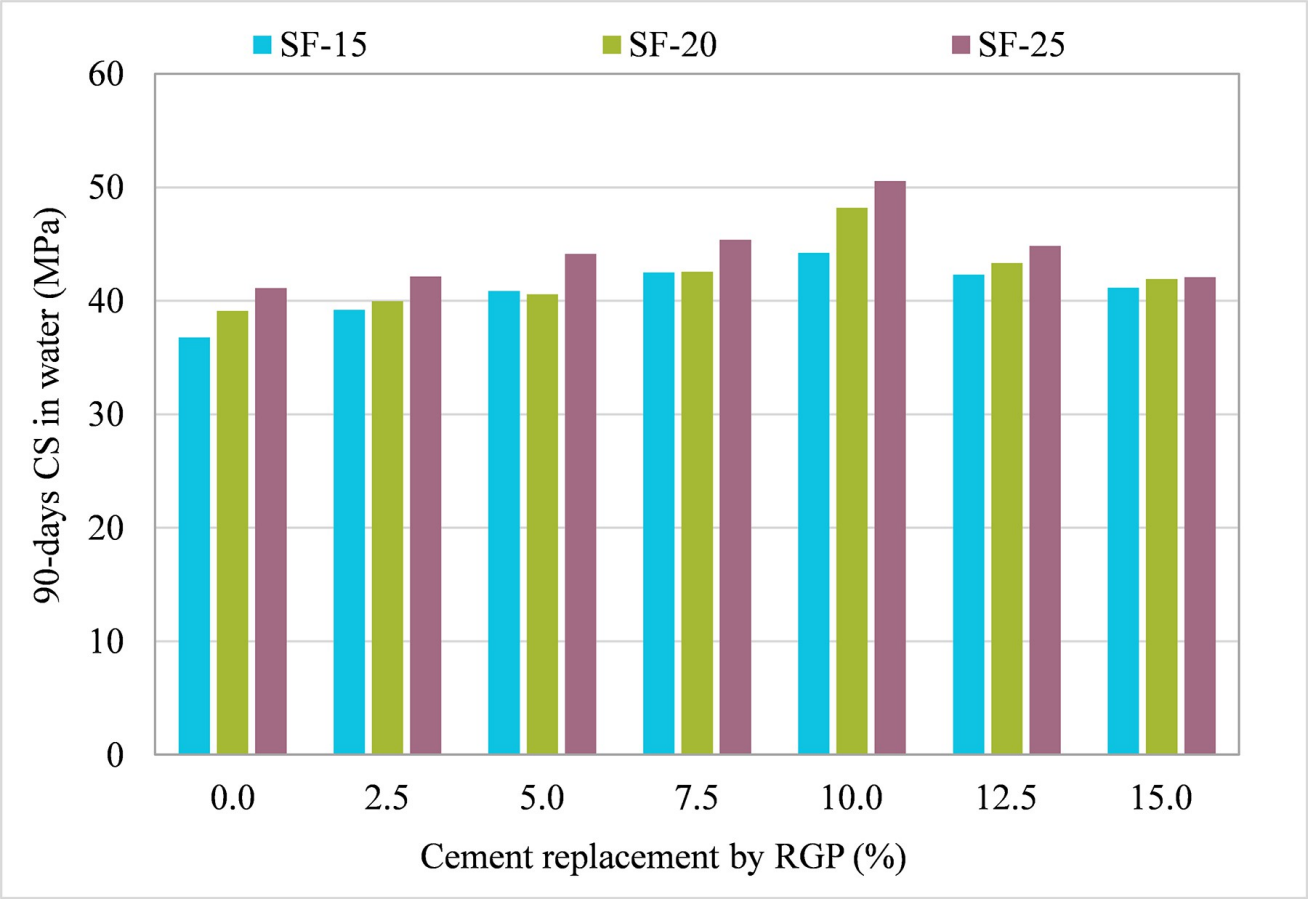
90-day CS of samples incorporating RGP as cement replacement in plain water.

Fig 6 displays the 90-day CS for samples that used RGP in place of cement. Increasing 90-day CS with RGP was shown to be possible with RGP usage. All mixes (SF-15, SF-20, and SF-25) resulted in a rise in 90-day CS of specimens at RGP ratios of up to 10% of cement, but then a decline in CS was noted. Nonetheless, the 90-day CS was higher than the control mix (CM) up to a ratio of 15% RGP. For instance, when comparing the CM to the SF-15 mix with RGP concentrations of 2.5%, 5%, 7.5%, 10%, 12.5% and 15%, the 90-day CS increased by 6.66%, 11.15%, 15.56%, 20.29%, 15.04%, and 11.91%, respectively. Similar findings were also found in other combinations (SF-20 and SF-25). Maximum 90-day CS was seen in both the SF-15 and SF-25 samples when using 10% RGP as a cement replacement; these values were almost 23.19% and 23.01% higher than the CM, respectively. The filler effect and pozzolanic characteristics of WGP are probable causes [31]. Because of the filler action, the void ratio was reduced, and the overall structure became more compact. Increased SiO_2_ in the glass’s composition [67] reacted with Ca(OH)_2_ in the cement matrix to generate an enhanced CSH gel, which in turn enhanced the strength of the cement mortar [68, 69]. The CS dropped at RGP concentrations of 12.5% and 15% because these mixes included more RGP than was necessary to ensure adequate pozzolanic activity and cement dilution [31]. Therefore, using RGP as a cement alternative of up to 10% is beneficial for achieving the greatest strength. Using up to 15% RGP as a cement substitute in cement mortar is possible without compromising the CS, producing eco-friendly and economically favorable material.

**Fig 7:**
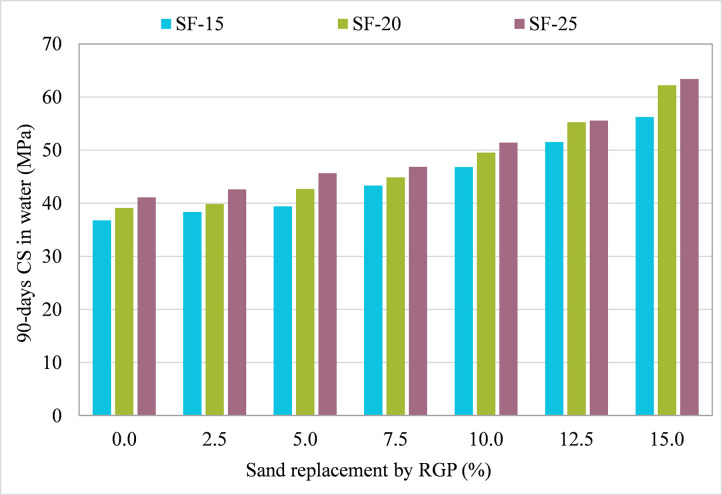
90-day CS of samples incorporating RGP as sand replacement in plain water.

Fig 7 depicts the outcomes of tests conducted on specimens with RGP replaced for sand. The 90-day CS rose when RGP was added to the three combinations (SF-15, SF-20, and SF-25) compared to the CM. With increasing RGP percentage, the 90-day CS increased, with the greatest increase seen at 15% sand replacement for all mixtures. In contrast to the CM, the 90-day CS of samples containing RGP at 2.5%, 5%, 7.5%, 10%, 12%, and 15% concentrations in the SF-15 mixture increased by 4.27%, 7.13%, 17.84%, 27.30%, 40.11%, and 52.93%, respectively. Similarly, the greatest increase in 90-day CS was attained with 15% RGP as a sand substitute in SF-20 and SF-25 mixes, which were 59.14% and 54.17% higher than the CM, respectively. Since RGP was finer than sand and resulted in enhanced particle packing, it is possible that this is the primary cause of the improvement in 90-day CS [70]. In addition, the use of RGP created superior hydration products (CSH gel) due to pozzolanic activity, which increased the 90-day CS of samples [31]. In order to get the maximum CS, 15% RGP might be utilized as a sand substitution. Moreover, it can be stated that utilizing RGP as a sand substitute is more advantageous than utilizing it as a cement replacement, given the rise in specimen 90-day CS.

### 3.2 Influence of RGP on 90-day CS in the acid solution

**Fig 8:**
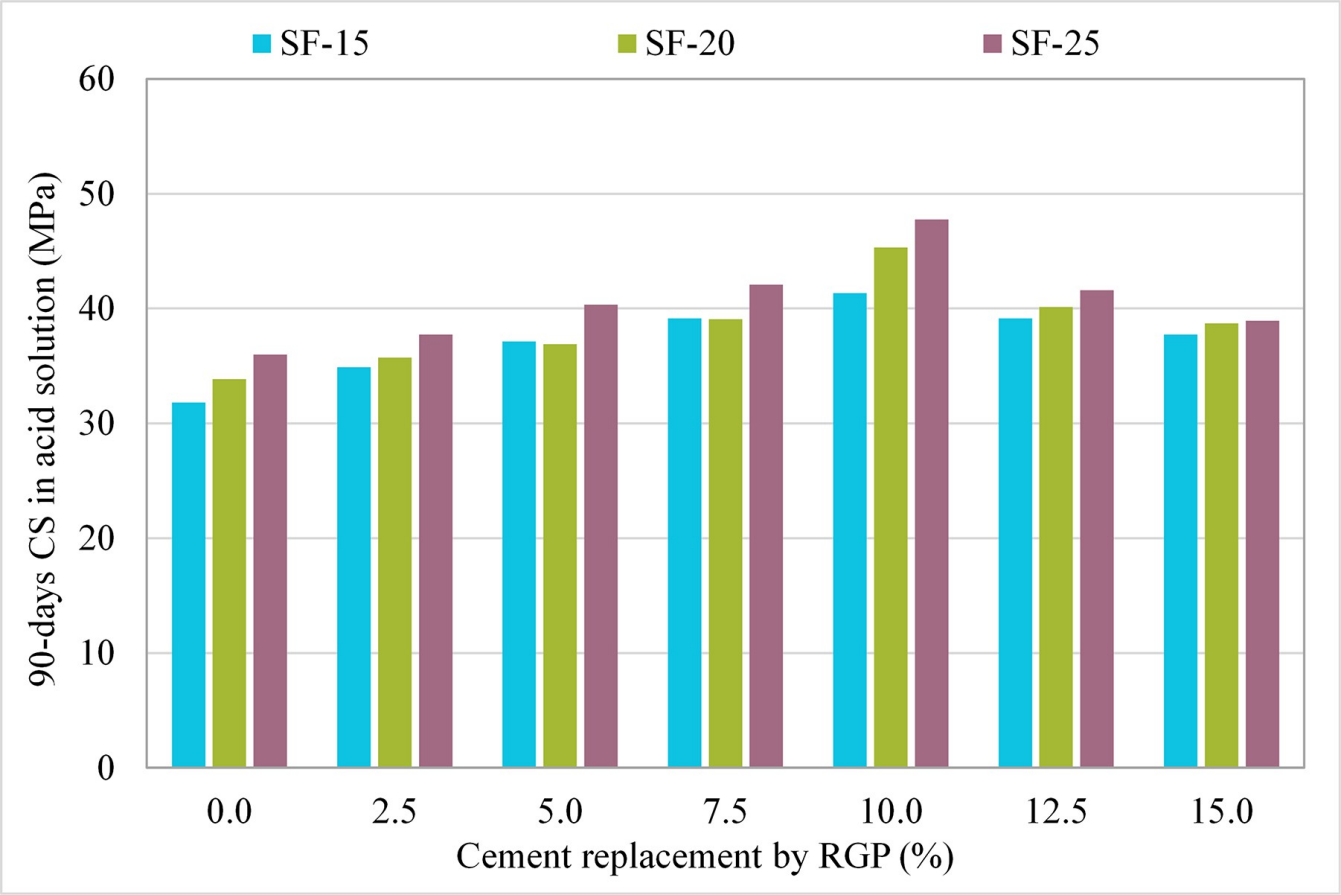
90-day CS of samples incorporating RGP as a cement replacement after subjected to the acid solution.

Results of the CS of acid-treated specimens that had RGP as a cement replacement are shown in Fig 8. It was noted that the samples containing RGP had greater resilience to the acid assault than the CM (0% RGP). The percentage difference in CS between each mixture and the CM treated to the same acidic condition was calculated. In all three mixtures (SF-15, SF-20, and SF-25), samples with 10% RGP as a cement replacement had the greatest CS following the acid attack. However, the CS of specimens after the acid attack was greater than the CM with up to 15% RGP content as a cement replacement in all mixes. For instance, at RGP ratios of 2.5%, 5%, 7.5%, 10%, 12.5%, and 15%, the CS of samples exposed to acidic solution in the SF-15 mix was enhanced by 9.65%, 16.72%, 23.04%, 29.93%, 23.07%, and 18.70%, respectively, compared to the CM. The highest CS following acid assault was found in SF-20 and SF-25 mixes with 10% RGP content, which was 33.90% and 32.73% higher, respectively, than in CM that had been exposed to the same acidic situation. As the higher Ca(OH)_2_ in the matrix is more susceptible to the acid attack [71] was consumed due to the presence of higher SiO_2_ content in RGP to form dense CSH gel [68, 69], hence, improving the acid resistance of the matrix.

**Fig 9:**
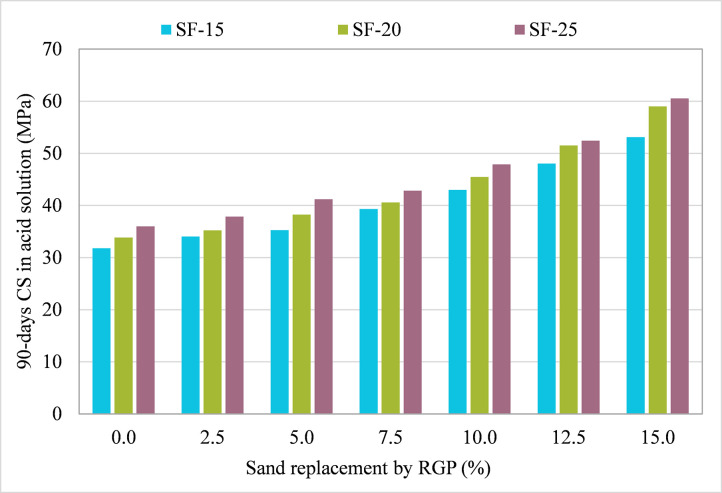
90-day CS of samples incorporating RGP as a sand replacement after subjected to the acid solution.

Fig 9 depicts the CS of specimens integrating RGP as a sand replacement after being exposed to the acid attack. In all combinations, 15% RGP as sand replacement yielded the highest CS following the acid attack. In the SF-15 mixture, the CS after the acid attack was found to be greater than the CM by 7.04%, 10.85%, 23.58%, 35.18%, 51.05%, and 66.99%, respectively, at RGP contents of 2.5%, 5%, 7.5%, 10%, 12.5%, and 15%. The greatest CS following the acid attack in SF-20 and SF-25 was seen at 15% RGP as a sand replacement, which was approximately 74.22% and 68.21% higher than the CM, respectively. The consumption of Ca(OH)_2_ due to the presence of SiO_2_ in RGP in order to create CSH gel in the matrix [68, 69] increased acid resistance. Therefore, it is advantageous to utilize RGP in cement-based materials in a harsh environment. In addition, the use of RGP as a sand substitute is more favorable than the use of a cement substitute due to its greater tolerance to acidic environments.

### 3.3 90-day CS comparison in plain water and the acid solution

#### 3.3.1 RGP as a cement substitute

**Fig 10:**
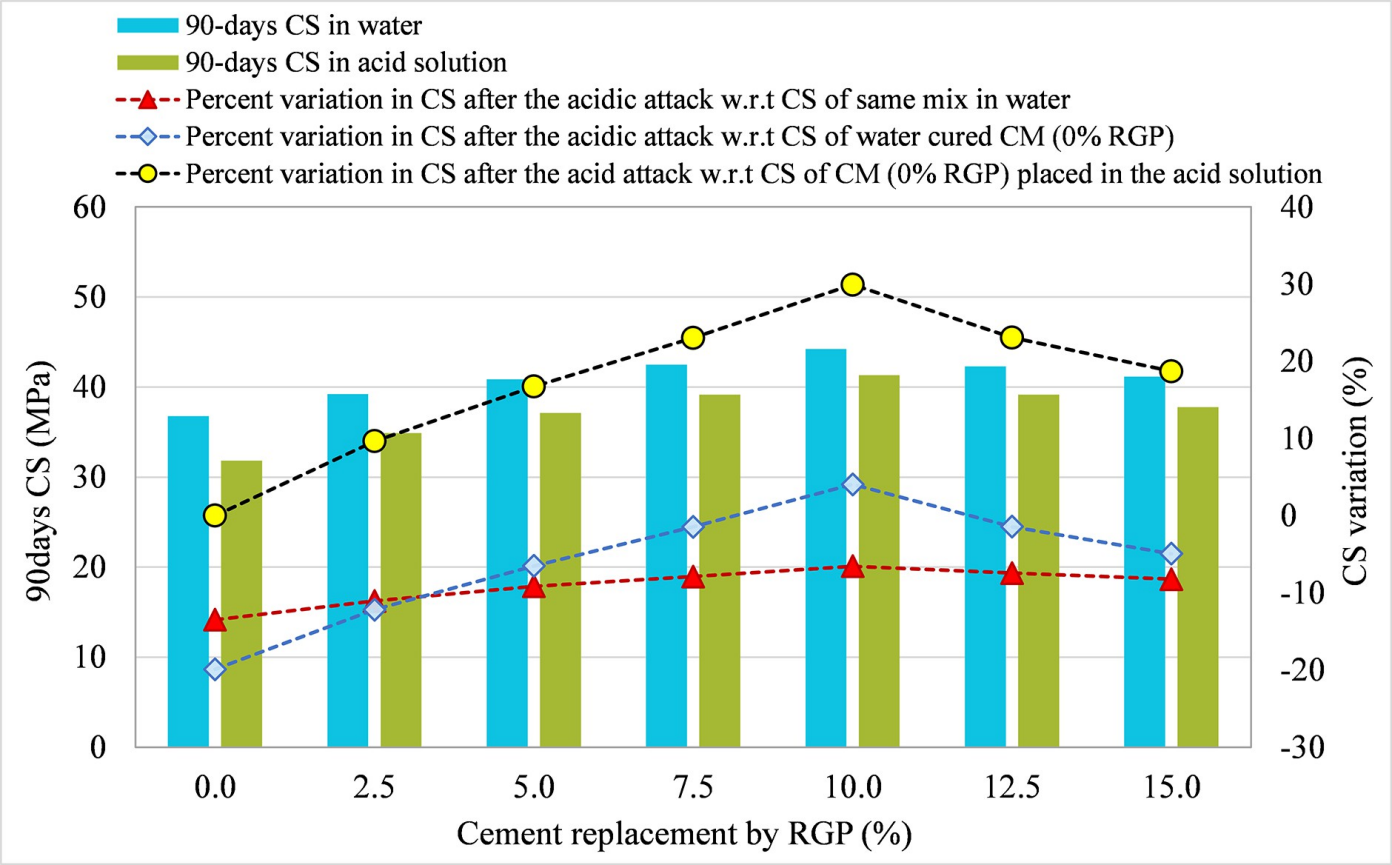
CS variation in samples placed in plain water and the acid solution for SF-15 mix incorporating RGP as a cement replacement.

**Fig 11:**
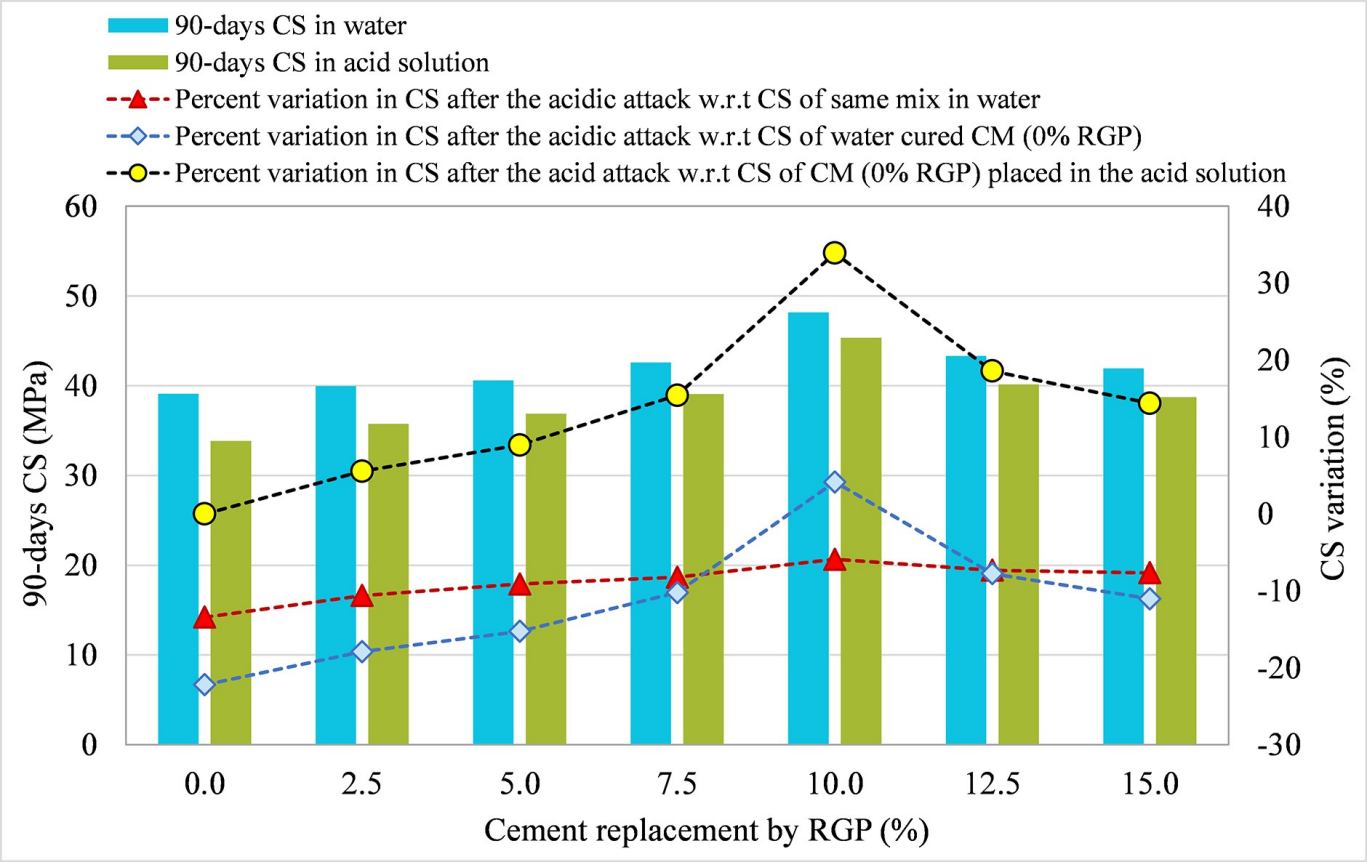
CS variation in samples placed in plain water and the acid solution for SF-20 mix incorporating RGP as a cement replacement.

**Fig 12:**
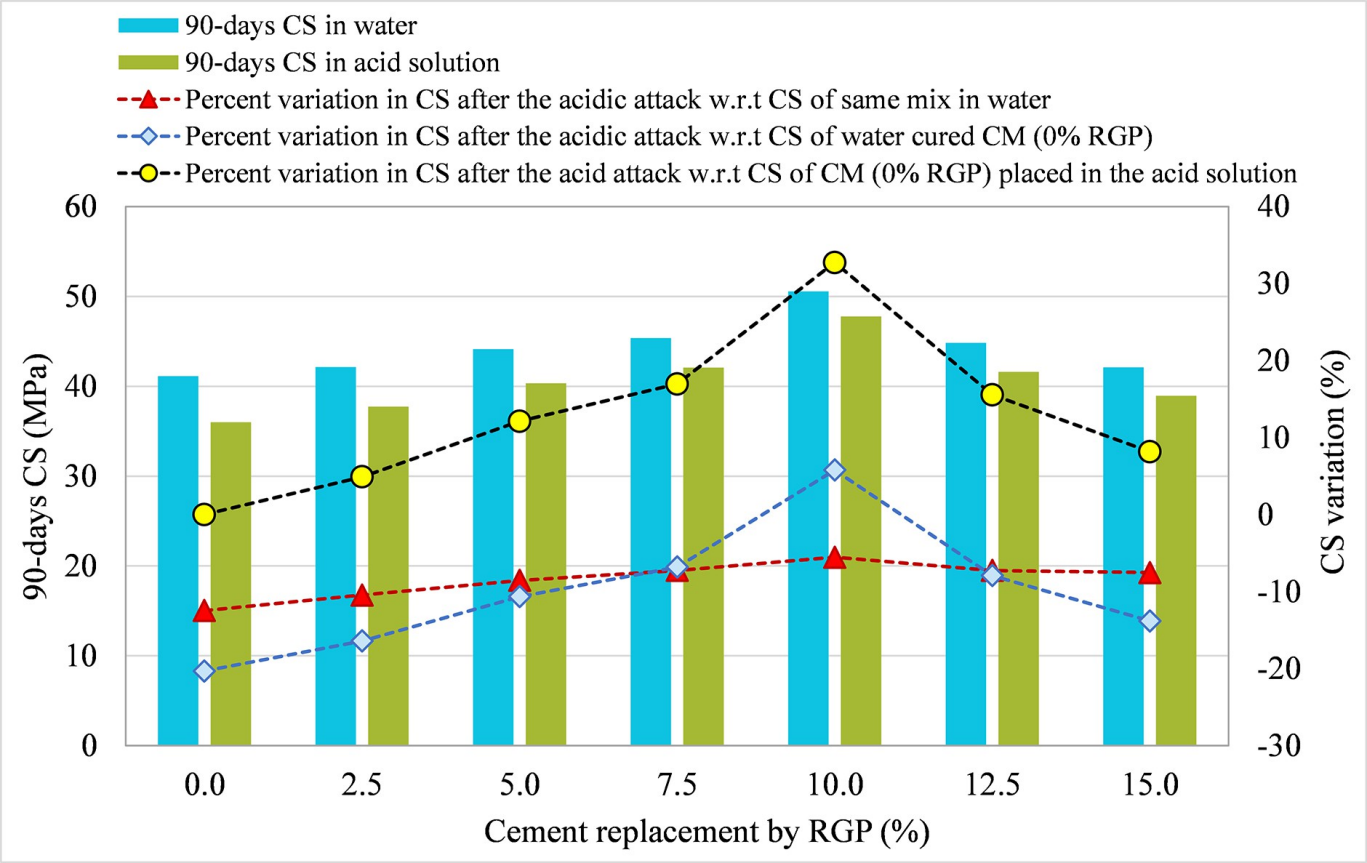
CS variation in samples placed in plain water and the acid solution for SF-25 mix incorporating RGP as a cement replacement.

For specimens integrating RGP as a cement replacement, 90-day CS in plain water and the acid solution were compared for all mixtures, and the results are shown in Figs 10–12 for the SF-15, SF-20, and SF-25 mixes, respectively. The figures illustrate the percentage change in CS following the acid assault calculated in relation to the 90-day CS of the same mix placed in water, the 90-day CS of CM placed in water, and the 90-day CS of CM submerged in an acidic solution. Comparing the SF-15 mix to the same mix poured in plain water, the CS dropped by 13.49%, 11.07%, 9.15%, 7.88%, 6.56%, 7.45%, and 8.24% at RGP ratios of 0%, 2.5%, 5%, 7.5%, 10%, 12.5%, and 15%, respectively (refer Fig 10). Similar findings were found in other mixtures, where samples with 10% RGP as a cement substitute showed the least drop in CS following the acid assault. After the acid assault on CMs with 0% RGP in the SF-20 and SF-25 mixes, the CS decrease was 13.42% and 12.45%, respectively. While the reduction in CS following the acid attack was 5.89% and 5.54%, respectively, in samples incorporating 10% RGP as a cement replacement in SF-20 and SF-25 mixes (refer Figs 11 and 12). It was discovered that specimens containing 10% RGP as a cement replacement had a higher CS than the CM put in water, with values that were around 4.05%, 4.16%, and 5.78% higher in SF-15, SF-20, and SF-25 mixtures, respectively. In all combinations, the CS was lower than the CM placed in water at the higher replacement levels of RGP. Similar to this, the highest resistance to acid attack was recorded at 10% RGP content as a cement substitute when the variation in CS after the acid attack was analyzed with regard to the CM treated in the same acidic environment. Therefore, given the greater resilience to aggressive conditions, the research findings support the use of RGP at a replacement level of 10% as a cement alternative.

#### 3.3.2 RGP as a sand substitute

**Fig 13:**
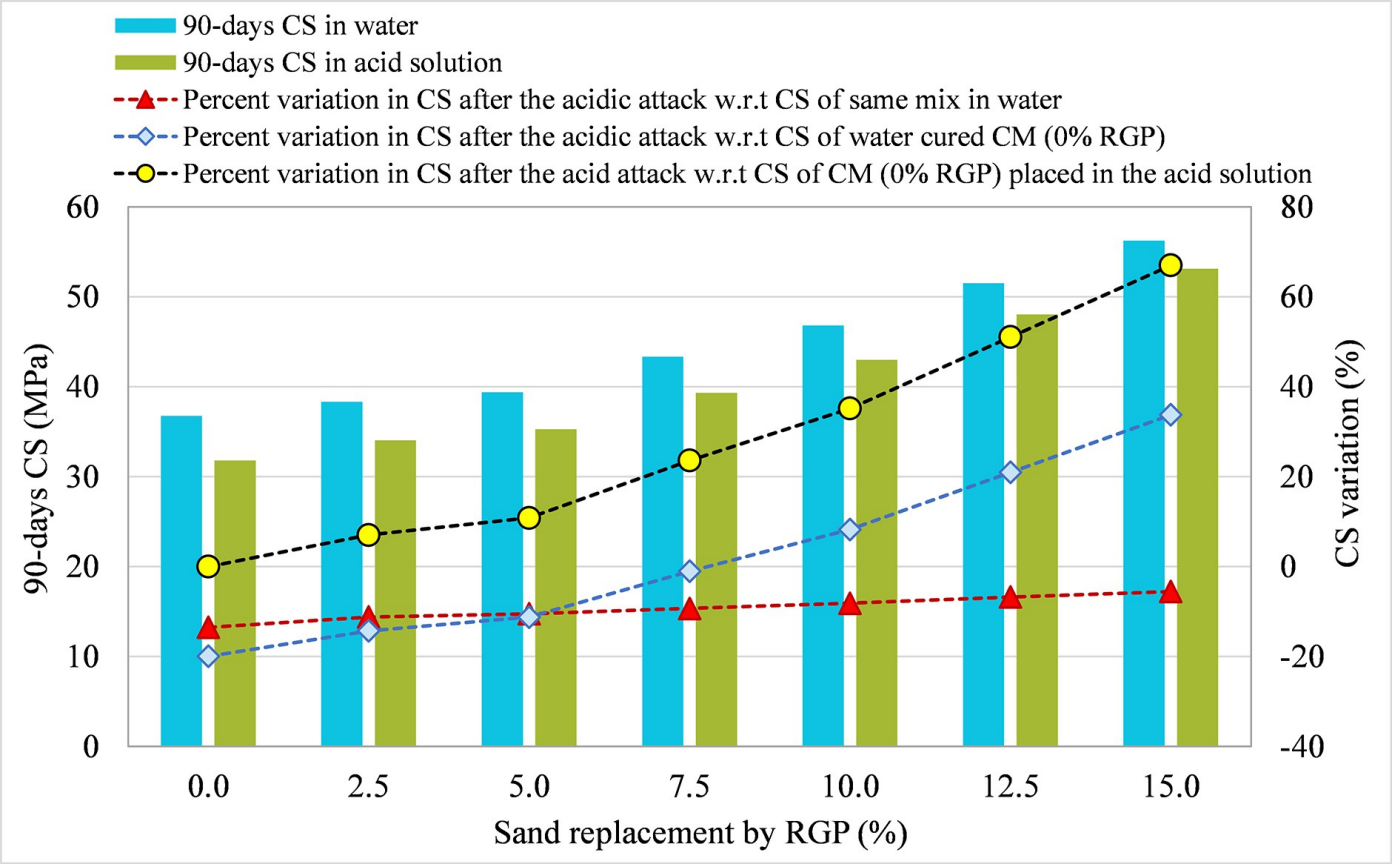
CS variation in samples placed in plain water and the acid solution for SF-15 mix incorporating RGP as a sand replacement.

**Fig 14:**
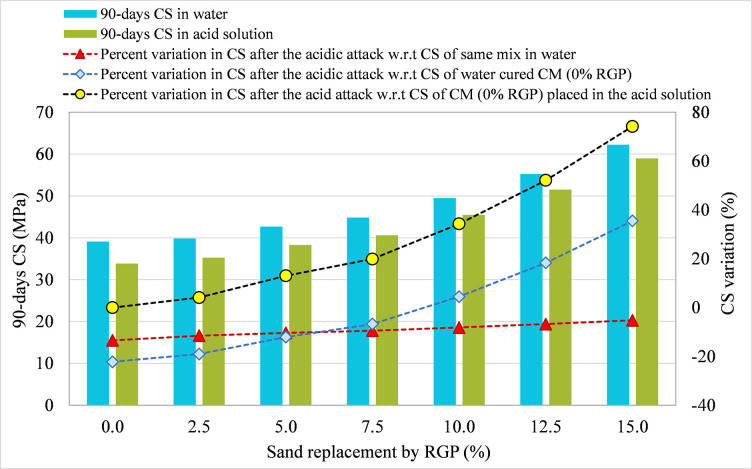
CS variation in samples placed in plain water and the acid solution for SF-20 mix incorporating RGP as a sand replacement.

**Fig 15:**
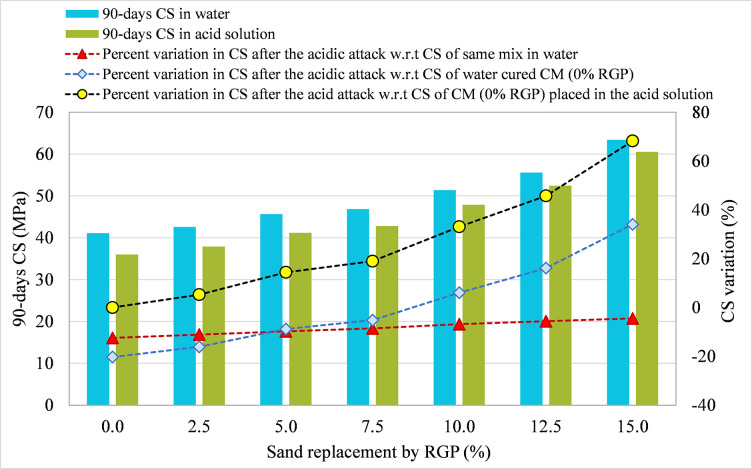
CS variation in samples placed in plain water and the acid solution for SF-25 mix incorporating RGP as a sand replacement.

Figs 13–15 show the comparison of the 90-day CS in plain water and acidic solution for specimens incorporating RGP as a sand substitute for mixes SF-15, SF-20, and SF-25, respectively. The percentage change in CS after an acid attack was determined in comparison to the CS after 90 days of the same combination immersed in water, CM submerged in water, and CM subjected to the acid solution. At RGP ratios of 0%, 2.5%, 5%, 7.5%, 10%, 12.5%, and 15%, the SF-15 mixture’s CS dropped by 13.49%, 11.19%, 10.48%, 9.28%, 8.14%, 6.74%, and 5.53%, respectively, compared to the same mixture stored in plain water (refer Fig 13). The 15% RGP sand replacement combination showed the least CS reduction after an acid attack, which was also found in other mixes. Samples using 15% RGP as a sand replacement in SF-20 and SF-25 mixes showed smaller decreases in CS by 5.22% and 4.48%, respectively, after the acid assault (refer to Figs 14 and 15). Moreover, in all three mixes (SF-15, SF-20, and SF-25), the CS of the specimens following acid assault containing 10%, 12.5%, and 15% RGP as a sand substitute was greater than the CS of the CM kept in water. After the acid assault, the CS of the CM in water was higher than the CS of the samples with RGP concentrations of 2.5%, 5%, and 7.5%. As an example, the CS of SF-15 mix samples that included 0%, 2.5%, 5%, and 7.5% RGP as a sand substitute following the acid attack was nearly 19.91%, 14.27%, 11.23%, and 1.03% lower than the CM kept in water. However, at 10%, 12.5%, and 15% RGP ratios, the CS was higher than the CM placed in plain water by nearly 8.26%, 20.97%, and 33.74%, respectively. Similarly, the CS of SF-20 and SF-25 specimens with 15% RGP as a sand replacement was greater than the CM kept in water by around 35.52% and 34.06%, respectively. The maximum resistance to acid attack was found at 15% RGP concentration as a sand substitute when comparing the variance in CS after an acid assault to CM treated in the same acidic environment. Thus, these results provide evidence in favor of using RGP as a sand substitute at replacement levels of 10–15% due to its resistance to acidic conditions.

### 3.4 Developed ML models

#### 3.4.1 Bagging regressor

**Fig 16:**
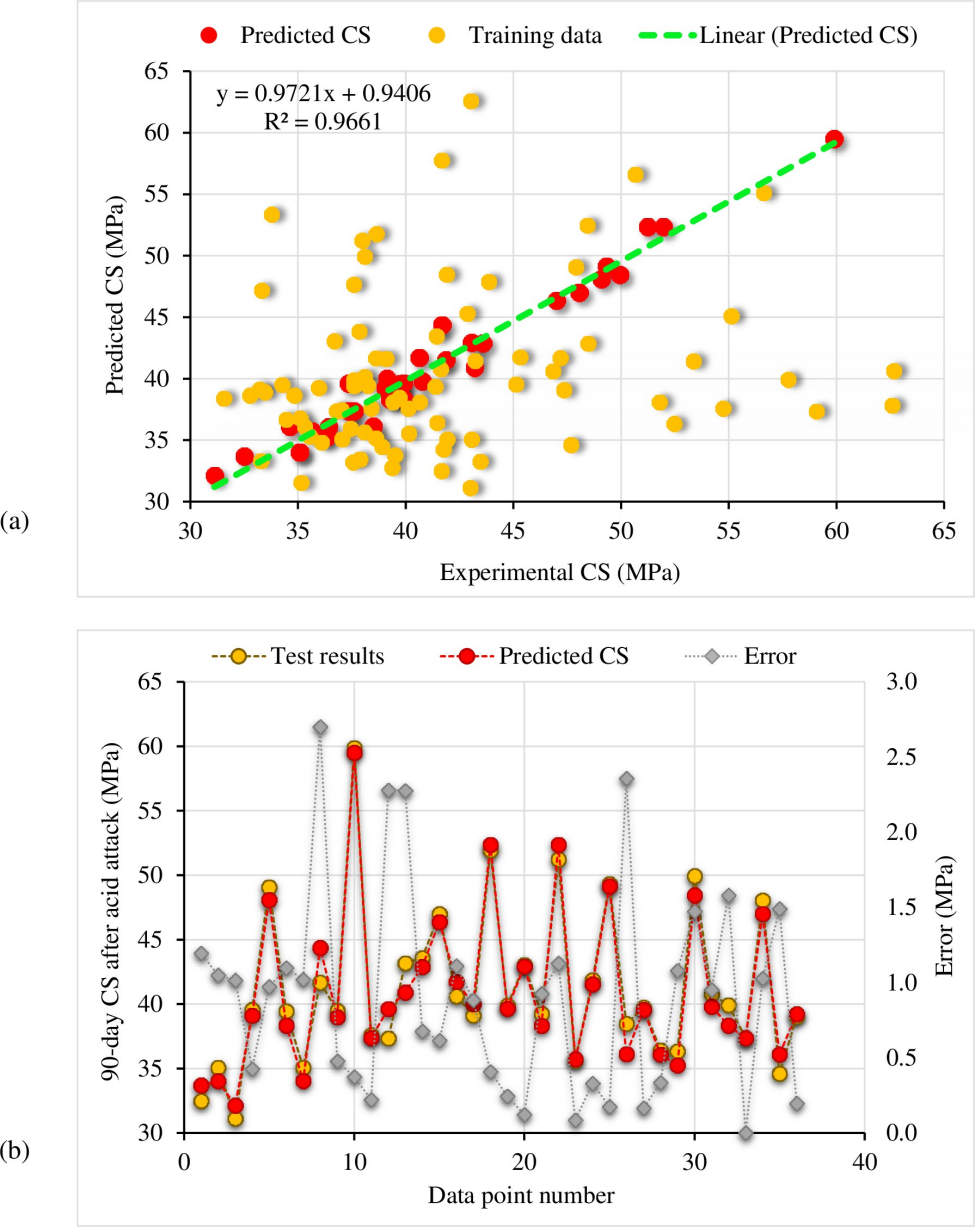
Bagging regressor model: (a) Correlation amongst experimental and predicted CS after the acid attack; (b) Representation of experimental, predicted, and error values.

Fig 16 shows the CS of cement mortar containing RGP determined using the bagging regressor approach following the acid assault. Fig 16(A) shows the correlation between the experimental and predicted CS. The bagging regressor model successfully predicted the findings with just a little discrepancy between the experimental and expected CS. The R^2^ value of 0.96 indicates a very strong concordance between experimental and projected results. Fig 16(B) shows the distribution of the experimental, predicted, and error values for the bagging regressor model. A maximum of 2.70 MPa discrepancy was detected, with an average of 0.90 MPa. In addition, the error values were analyzed proportionally, revealing that for 20 samples, the error was less than 1 MPa; for 12 samples, the error was between 1 and 2 MPa; and for 4 samples, the error was greater than 2 MPa. The distribution of divergence data showed that the CS of cement mortar following exposure to an acidic environment might be accurately predicted using a bagging regressor model.

#### 3.4.2 Random forest

**Fig 17:**
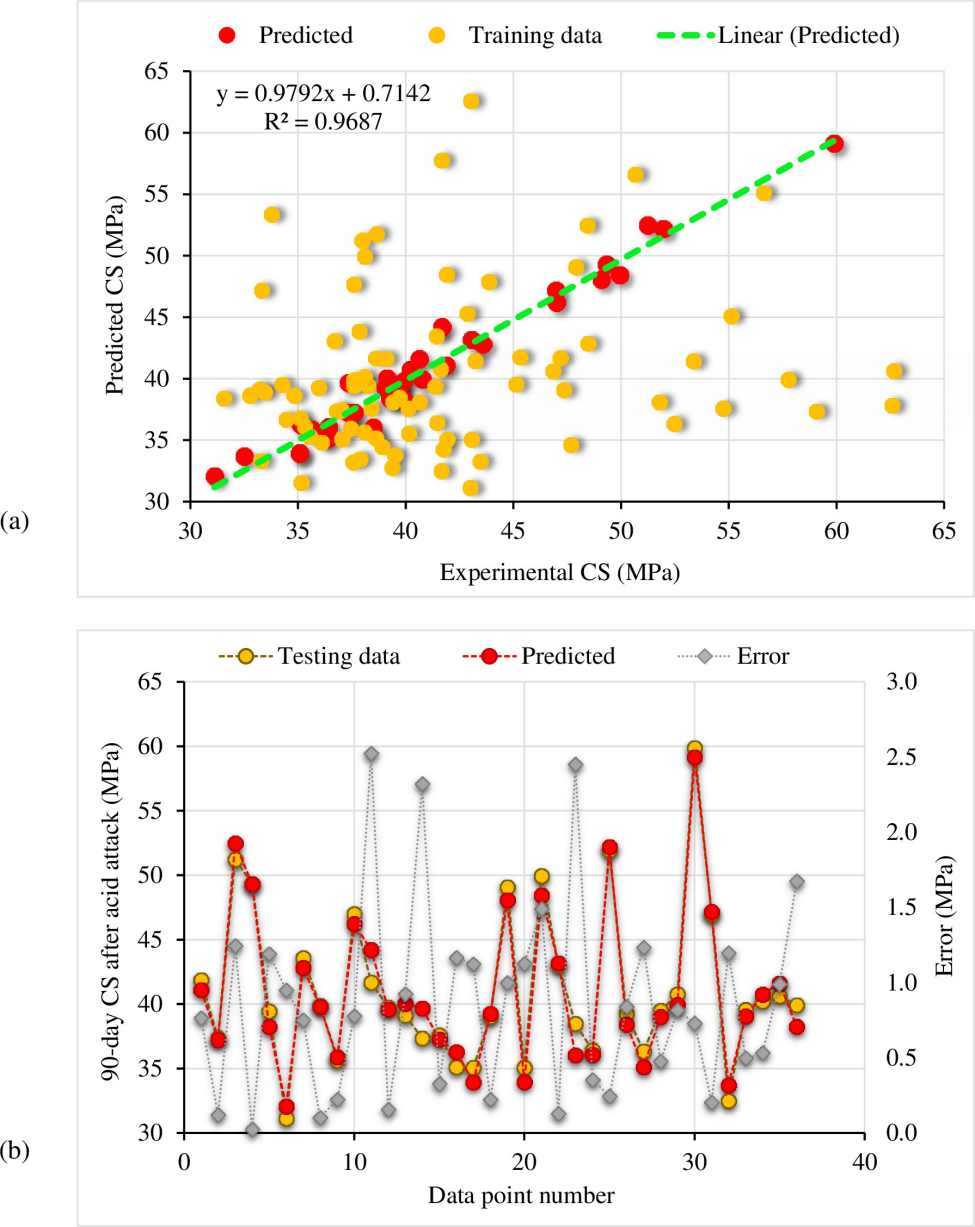
Random forest model: (a) Correlation amongst experimental and predicted CS after the acid attack; (b) Representation of experimental, predicted, and error values.

The random forest approach outcomes to predict the CS of the RGP-based cement mortar after the acid assault are depicted in Fig 17. The correlation between predicted and experimental CS is shown in Fig 17(A). The random forest model had a smaller gap between the experimental and projected CS than the bagging regressor model. The higher R^2^ value of 0.97 for the random forest model demonstrated its greater accuracy. The distribution of actual, estimated, and error values for the random forest method are shown in Fig 17(B). Maximum error was determined to be 2.52 MPa, with an average of 0.86 MPa. Furthermore, 24 data samples had error values below 1 MPa, 9 data samples had error values between 1 and 2 MPa, and only 3 data samples had error values beyond 2 MPa. As the error distribution shows, the random forest model is more precise than bagging regressors. In the random forest training procedure, each tree generates regression, and the forest with the most votes is chosen as the model, resulting in increased model accuracy [50]. However, the results of the bagging regressor model were also found to be accurate. Thus, both employed models can be used to predict the CS of cement mortar after the acid attack.

### 3.5 ML model’s validation

The results of statistical tests (MAE, RMSE, and MAPE) used to evaluate the errors are summarized in Table 1. The MAE for the bagging regressor model was 0.903 MPa, whereas, for the random forest model, it was 0.859 MPa. Among the two ML models used, the bagging regressor MAPE was 2.30%, and the random forest MAPE was 2.20%. In addition, the RMSE for the bagging regressor was 1.133 MPa, whereas that for the random forest was 1.071 MPa. It was clear from these evaluations that the two models used had similar levels of accuracy. Results for the k-fold technique, including estimates of R^2^, RMSE, and MAE, are shown in Table 2. The bagging regressor MAE was 1.19 MPa, ranging from 0.27 MPa to 3.61 MPa. The MAE for the random forest model was 1.16 MPa, ranging from 0.16 MPa to 3.44 MPa. The RMSE for the random forest model was 1.48 MPa, whereas the RMSE for the bagging regressor model was 1.51 MPa. The average R^2^ values for the bagging regressor and random forest models were 0.82 and 0.84, respectively. Both the bagging regressor and random forest models, with their lower error rates and better R^2^ values, were shown to have equivalent accuracy when estimating the CS of cement mortar containing RGP after the acid attack.

**Table 1:** Results of statistical checks for the ML models.

ML model	MAE (MPa)	MAPE (%)	RMSE (MPa)
Bagging regressor	0.903	2.30	1.133
Random forest	0.859	2.20	1.071

**Table 2:** Results of the k-fold validation method.

K-fold	Bagging regressor			Random forest		
	MAE	RMSE	R^2^	MAE	RMSE	R^2^
1	0.54	0.53	0.96	0.49	0.58	0.97
2	0.80	0.83	0.95	1.02	1.09	0.97
3	1.38	1.77	0.84	1.24	1.64	0.88
4	3.61	4.87	0.80	3.44	4.71	0.81
5	0.66	1.03	0.94	0.93	1.07	0.97
6	1.22	1.63	0.83	1.21	1.49	0.84
7	0.27	0.43	0.93	0.16	0.14	0.96
8	1.24	1.49	0.44	1.23	1.50	0.49
9	1.68	2.05	0.67	1.33	2.06	0.64
10	0.46	0.50	0.89	0.52	0.55	0.90

### 3.6 Relevance of raw ingredients

**Fig 18:**
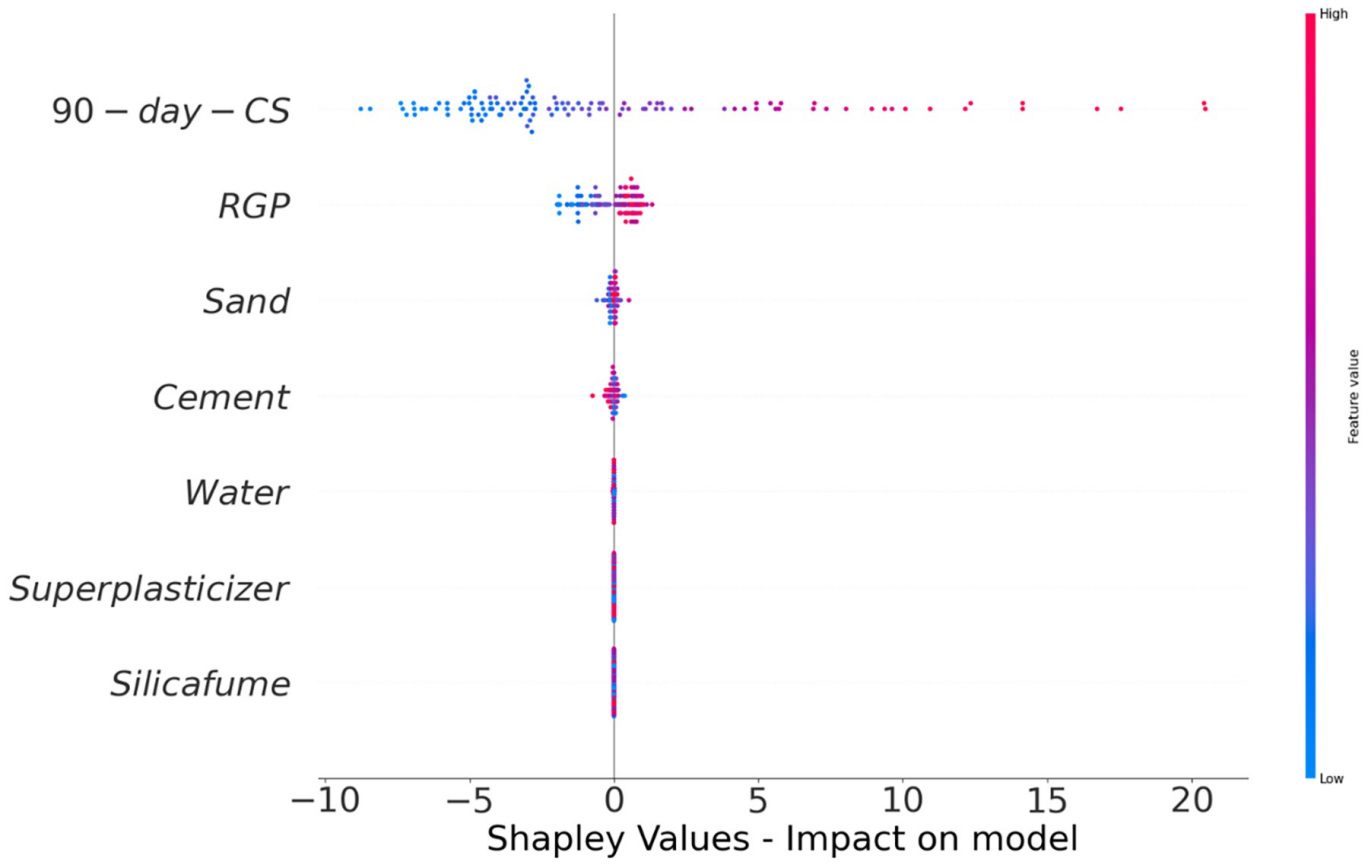
Relevance of raw components on the CS after the acid attack.

Using SHAP analysis, this research examined the effects of raw components on the CS of cement mortar following the acid attack. A SHAP tree explainer was utilized to explore a deeper understanding of both the local SHAP explanations and the global feature propositions of the dataset. Fig 18 depicts the results of the violin SHAP plot for the input parameters demonstrating their influence on CS after the acid attack. On the x-axis of the graph, the contribution of each raw material is shown as a SHAP value, and each variable value is represented by a distinct color. The 90-day CS of cement mortar was found as the most significant variable in the dataset with a greater positive influence (more red points on the right side), showing that the higher the 90-day CS of a material, the greater its resistance to acid assault. The second most crucial component was determined to be RGP, with a more positive impact on the CS of cement mortar after the acid attack. However, there exists an optimal limit beyond which the incorporation of RGP results in the negative impact on CS of cement mortar after the acid attack, as evidenced by the experimental findings when RGP was used as a cement replacement. Hence, it may be advantageous to utilize RGP to an optimal percentage in order to prevent CS loss following an acid assault. In addition, both positive and negative impacts of sand and cement were seen, indicating that the sand and cement contents should be maintained within optimal ranges in order to manage the CS loss of cement mortar after the acid assault. Due to the decrease in data variance, the effects of SF, superplasticizer, and water were less definite. A bigger data sample with a more range of input parameters might give more trustworthy findings.

## 4. Discussions

This work employed both experimental and modeling methods to explore how exposure to an acidic environment influenced the CS of cement mortar in which RGP was utilized as a partial replacement for sand and cement. Cementitious composites are widely used building materials [72–75]. Globally, the present research focuses on producing eco-friendly building materials [76–78]. The majority of the world’s waste glass is dumped in landfills, which causes threats to human health and the environment [31]. Because of their widespread usage, cement-based materials contribute to the depletion of natural resources and the emission of carbon dioxide. The substitution of RGP for cement and fine aggregate in cement-based materials may be a beneficial approach. As a result, the use of RGP in cement-based materials will mitigate the negative effects on the environment by waste prevention, conservation of natural raw resources, and decreased carbon dioxide emissions.

The purpose of this research is to gain a better understanding of the use of RGP in cement mortar under harsh conditions by combining experimental and ML-based modeling techniques. Using a range of 0–15% RGP as a sand and cement replacement, mortar samples were tested in a 5% H_2_SO_4_ solution. Based on experimental results, it was shown that RGP incorporation controlled the decrease in CS following the acid attack. The smallest reduction in CS was observed when RGP was replaced for cement at a rate of 10%, with a drop of 5.54% compared to the same mix put in plain water. In addition, it was shown that specimens with 10% RGP as a cement substitute had a higher CS by up to 5.78% than the CM placed in water. The greater SiO_2_ content in RGP was used to produce thick CSH gel [68, 69], which enhanced the acid resistance of the matrix by consuming Ca(OH)_2_ in the matrix, which is more susceptible to acid attack [71]. At higher replacement levels as cement substitute, more RGP was used than was required for the pozzolanic reaction and cement dilution, leading to a higher CS loss [31]. For optimum resistance to an acidic environment, it is proposed that up to 10% RGP be used as a cement alternative. Using RGP as a sand replacement in cement mortar resulted in a decline in CS of up to 4.48% at a 15% RGP ratio following acid assault, compared to the same mix put in plain water. Moreover, the CS of the specimens after acid assault with 10%, 12.5%, and 15% RGP as a sand substitute was higher than the CS of the CM kept in water, with the 15% RGP samples giving the greatest resistance to the acid attack, resulting in up to 33.74% increase in CS compared to the CM in plain water. Consequently, RGP may be employed at a concentration of up to 15% as sand substitute to strengthen cement mortar against acid assaults. However, additional study is needed to assess RGP’s usefulness at higher replacement levels. Further, the lesser drop in CS of samples after acid attack indicated that the inclusion of RGP as a sand replacement was more valuable than its utilization as a cement replacement.

The tests data was systematized to develop ML models. Estimating the CS of the RGP-containing cement mortar following the acid assault was accomplished using two ML techniques: bagging regressor and random forest. Based on a thorough analysis of ML techniques in terms of accuracy, the optimal predictor was determined. A comparable accurate performance was noted between the two models in terms of R^2^ value, discordance among experimental and predicted outcomes and error assessment. Previous research has demonstrated the accuracy of the bagging regressor and random forest approaches for assessing the qualities of building materials [65, 79–82]. It is challenging to identify and propose the optimum ML method for predicting outcomes in diverse fields of study since the effectiveness of an ML technique is primarily dependent on the amount of input parameters and datasets used to run algorithms. The weak learner is used again and over again in an ensemble ML approach, which creates submodels that are trained on the dataset and adjusted to modify the R^2^ value. As a result, the final findings are more precise than those of the single ML models [20]. Therefore, this research suggests the use of both bagging regressor and random forest for predicting the CS of materials after the acid attack. Since ML research applications can speed up the development of fast and low-cost methods for determining material attributes, the construction industry might benefit from their use.

## 5. Conclusions

Experimental testing and machine learning (ML) modeling research was conducted to investigate the influence of incorporating recycled glass powder (RGP) in a cement mortar after being subjected to an aggressive environment. After the experimental testing, the generated data was used to develop two ML models, including bagging regressor and random forest, to predict the compressive strength (CS) of cement mortar subjected to the acid attack. The study’s main conclusions are given below:

- The maximum 90-day CS of cement mortar was achieved when RGP was used as a cement and sand replacement of 10% and 15%, respectively. The possible causes for the rise in CS include the filler effect due to RGP’s finer particle size and the pozzolanic reaction due to increased SiO_2_ content in glass.
- RGP-modified cement mortar lost less CS after being exposed to the acidic solution. Using RGP as a cement and sand substitute lowered CS by up to 5.54% and 4.48%, respectively, compared to the same mix kept in plain water. The optimum content of RGP as cement and sand substitute was found to be 10% and 15%, respectively.
- The comparison of CS of RGP-modified mortar specimens after the acid attack to that control mix kept in plain water for 90-days showed that at 10% cement and 15% sand substitutes an increase in CS of up to 5.78% and 33.74%, respectively, compared to the control mix in plain water. Therefore, the incorporation of RGP as a sand substitute was determined to be more advantageous than its usage as a cement replacement.
- It was determined that the CS of RGP-modified cement mortar following the acid attack may be estimated using the established ML models, which agreed well with the test findings. The prediction performance of the bagging regressor and the random forest models were found to be likewise accurate based on the R^2^ value (bagging regressor: 0.96 and random forest: 0.97), the divergence between experimental and predicted outcomes, and errors analysis (MAE, RMSE, and MAPE).
- According to the results of the SHAP analysis, the 90-day CS of cement mortar and RGP were the most important factors with a significant positive influence on the CS of the material following the acid attack. However, an optimum limit for RGP existed beyond which the addition of RGP had a detrimental effect on the CS of cement mortar following the acid attack.
- By reducing the environmental problems caused by waste glass disposal, conserving natural resources, providing cost-effective materials, and reducing carbon dioxide emissions, the reuse of waste glass in the building would promote sustainable development.

Additional investigation is required to generate a dataset with wide variety of input factors, such as the physical and chemical properties of raw ingredients, water-to-cement ratio, and surrounding conditions (humidity and temperature), in order to construct ML prediction models and investigate their effect on cement mortar strength. These factors were not considered in the modeling phase of this study because these were constant across all blends tested.
