# Expression of Concern: Evaluating the compressive strength of glass powder-based cement mortar subjected to the acidic environment using testing and modeling approaches

**DOI:** 10.1371/journal.pone.0350699

**Published:** 2026-06-02

**Authors:** The PLOS One Editors

The *PLOS One* Editors issue this Expression of Concern because this article [1] was identified as one of a series of submissions for which we have concerns about the peer review process. Readers are advised to interpret the article [1] with caution.
